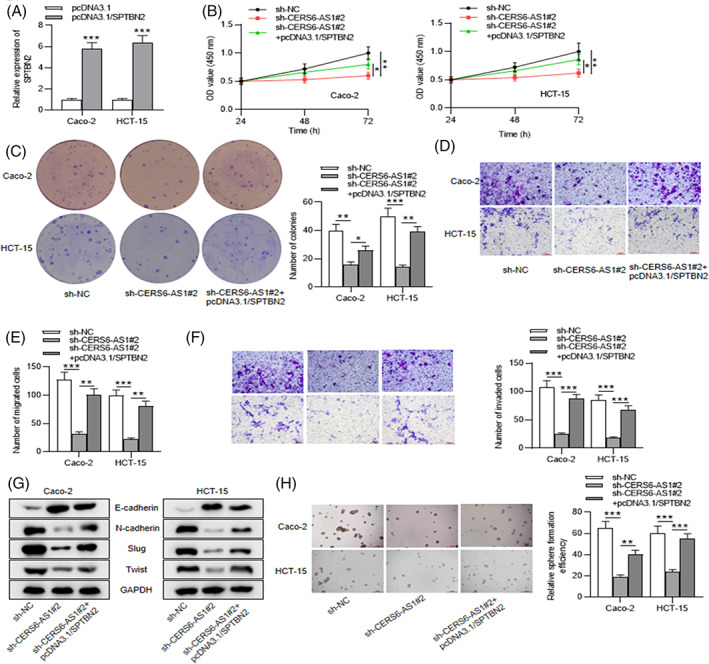# CORRIGENDUM

**DOI:** 10.1002/kjm2.12603

**Published:** 2022-10-10

In the manuscript “Zhao S‐Y, Wang Z, Wu X‐B, Zhang S, Chen Q, Wang D‐D, et al. CERS6‐AS1 contributes to the malignant phenotypes of colorectal cancer cells by interacting with miR‐15b‐5p to regulate SPTBN2. Kaohsiung J Med Sci. 2022;38:403–14. https://doi.org/10.1002/kjm2.12503”, the author has found errors in the Figures 2 and 5.

Some mistakes in the data for images showing the migratory and invasive abilities of Caco‐2 and HCT‐15 cells were found. Figure 2D, 2F, 5D, and 5F to be replaced are below, and this correction does not affect any of the conclusions of the article.

Revised Figure 2:

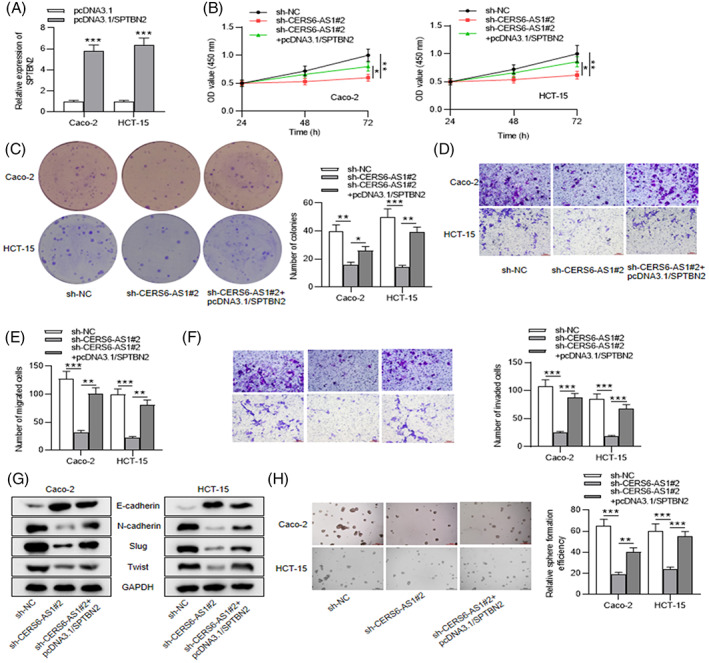

Revised Figure 5: